# The Education of the Dentist

**Published:** 1911-06-15

**Authors:** E. C. Kirk


### THE EDUCATION OF THE DENTIST

BY E. C. KIRK, D.D.S., D.S.C.

I think it was Prof. Black who said on one occasion, that the education of the dentist should begin at his conception. I would not take such primitive ground as that, but think it important to consider that his education should begin with the preparatory school. I want to say something to the teacher in the preparatory school, pointing out to him and to you my idea of the proper material from the product of the preparatory schools from which we may get our dentists. One criticism upon this product as a whole, is defective understanding of languages, and inability to analyze the same. The more I have to do with the product of High Schools and preparatory education, the more I am convinced of this great fault in that material. Experience shows us in the course of our professional training that from twenty-five to forty per cent. of the material we get is unfit, and we can easily see that something is going wrong. We are demanding a higher standing, and there is need of higher preparatory education for taking up professional course of instruction. In considering this question carefully, the defect in the product of the High Schools is due to not giving enough attention to the understanding of language and the meaning of words.

I read recently in a London paper the same complaint made by a gentleman from South Africa, dealing with educational matters there, that their great difficulty was the fcat that they attempted to teach the English language in close proximity with another language, and they experienced difficulty in endeavoring to keep the English language pure. We experience the same difficulty in the State of Pennsylvania, owing to the close proximity of many foreign speaking people.

In 1896, when I was asked to take charge of the dental school of the University of Pennsylvania, I was asked this question: "Is there any good reason why the status of the dental students of our University should be any different from any other department of the University?" I thought for a moment and said: "There is, and no, there need not be." The whole question was a question of the educational fitness of the men. As soon as you make the requirements for this department in the way of preparatory education, the same as any other department in the University, they will have the same status.

In the education of the dentist, assuming what I have said to be correct, we should have a well educated staff of men take up the practice of dentistry The extent of the education which he should receive in the dental school is another consideration.

We have been accustomed to secure our work from a purely mechanical standpoint. It would seem that dental mechanics has reached the point where it cannot be developed any further, because we have labored so strictly in that direction that other important questions before us have been neglected, we are still treating dental caries mechanically. Is that all we can do? Not withstanding the research work that has been done we are still attempting to treat dental caries by the same mechanical processes. What have we done for the human race and for the profession? We have got to do something along other lines. There are other questions to be solved, and in order to solve these problems as they should be we need educated men, and in order to get educated men we must have better preparation. I am opposed to the lengthening of the time of preparation, I want to ask secondary schools to do better work, I want a better kind of education, not a larger amount of it. I also want more time in the dental course for the education of dentists, I want at least a four-year course. We must not however think that the responsibility for such an advance rest wholly upon the dental colleges, it is not upon the schools, it is upon the members of the dental profession. When the sentiment of the dental profession is such that it will refuse to send its students to a school with a course less than four years, the necessary result will be accomplished.

I want also to make a plea for better teaching in dental schools, I make this plea for better teaching in dental schools just as I make the plea for better teachings in the preparatory schools. Teachers infused with the idea that teaching is worth while, that there is something more than the compensation he receives in dollars and cents are greatly to be desired to-day. We need more men who are fit to take up the work of teaching. It would be a difficult matter for me today, if I were to loose a member of our faculty, to fill the position made vacant, for most dentists are not fitted or inclined to take up teaching. We want men who are able to supply culture and a high standard in the work of the dental curriculum so that we may turn out material that will teach students to respect their work and calling.

I am constantly amused at the discussions about dental fitness. What is dental fitness? How does it differ from other fitness resulting from properly conducted education? I want to say to you men who are interested in general education that you have an important work to perform. I speak of primary and secondary education, and to those of you who stand for a longer course and higher standards in our schools. It is not necessary to enter into a discussion of what constitutes dental education with you. Use your energies along lines which will tend to the production of a class of men who are enthused with the idea that there is something more in all life callings than mere commercialism or mechanics for these are the lines of progress in all special education.

Incidental there will be a demand, and there is a demand today, for post-graduate work and further education of those who are to solve our great problems. We need an opportunity for men so inclined who have in the past received their education under conditions where these opportunities were not afforded. We are hoping that in the near future we shall have in the dental schools of the University just such a place in the new building. I hope that when that structure is completed we shall have facilities for post-graduatework and original research. In order to make these things particularly valuable and to make them of the most use we should start out with the idea that our calling is not largely one of mechanics, but that we have other obligations. I want to state that I have stood from the first for the idea that we should start out with the idea that we are going to make a dentist and that we are not going to make a mechanic. I want to stand for a larger and stronger curriculum so that students in dental schools shall be graduated not only with the degree of Doctor of Dental Surgery but with a broad and deep scientific and cultural basis as well.—*Scrap Book.*

